# Evaluation of Satisfaction and Outcomes of Patients Who Underwent Two-Piece Inflatable Penile Prosthesis Implantation

**DOI:** 10.7759/cureus.26097

**Published:** 2022-06-19

**Authors:** Yasar Pazir, Fatih Yanaral, Ufuk Caglar, Mazhar Ortac, Omer Sarilar, Faruk Ozgor

**Affiliations:** 1 Department of Urology, Haseki Training and Research Hospital, Istanbul, TUR; 2 Department of Urology, Istanbul Faculty of Medicine, Istanbul University, Istanbul, TUR

**Keywords:** partner satisfaction, two-piece inflatable penile prosthesis, erectile dysfunction, penile prosthesis, patient satisfaction

## Abstract

Introduction

The aim of this study was to evaluate patient and partner satisfaction, device reliability, and complications in patients who underwent two-piece inflatable penile prosthesis (IPP) implantation.

Patients and methods

The data of 22 patients who underwent two-piece inflatable penile prosthesis implantation in our department between 2015 and 2018 were retrospectively analyzed, and a detailed review of all clinical reports was performed. Phone or face-to-face interviews were undertaken to assess the satisfaction rates of the patients and their partners using the modified Erectile Dysfunction Inventory of Treatment Satisfaction (EDITS) questionnaire.

Results

The mean patient age and partner age were 57.95 ± 6.16 and 58.12 ± 6.66 years, respectively. The mean erectile dysfunction (ED) period was 5.33 ± 2.16 years, and the etiologies of erectile dysfunction were radical pelvic surgery (41%), diabetes mellitus (37%), and vascular disorders (22%). The mean operative time and postoperative hospital stay were 102 ± 29 minutes and 1.8 ± 0.66 days, respectively. Over a mean follow-up period of 29.04 ± 14.48 months, two (9%) cases underwent revision surgery due to mechanical device failure in one and infection in the other. The overall patient and partner satisfaction rates were 73% and 59%, respectively.

Conclusions

The two-piece inflatable penile prosthesis is an effective, reliable, and user-friendly prosthesis with acceptable complication and revision rates and provides a high level of patient and partner satisfaction in selected and fully informed patients.

## Introduction

A penile prosthesis is recommended as a third-line treatment modality for patients with erectile dysfunction (ED) who do not respond to conservative medical treatment or seek a permanent solution [1]. There are several types of penile prosthesis available on the market, including malleable, two- and three-piece inflatable devices. Today, although the three-piece inflatable prosthesis is the most preferred since it can achieve a more natural erection, it may not be ideal for every patient [2]. The device designed as two pieces has several advantages over three-piece prostheses, such as ease of implantation, low cost, and ease of use. Furthermore, for patients with limited manual dexterity, it is easier to activate and deactivate two-piece inflatable penile prostheses (IPPs) [3]. The lack of a need for separate reservoir placement is a further advantage that shortens the duration of the procedure, and it is a feasible option for pelvic organ transplant recipients or patients with a retropubic scar from previous surgery. It is also a suitable option for low-volume implanters due to its ease of implantation and low complication rates [4,5]. In addition, in some countries, three-piece IPPs are not covered by health insurance, leading patients to prefer two-piece alternatives due to economic reasons [6,7].

The main purpose of penile prosthesis implantation is to achieve high patient and partner satisfaction. However, the main concern of patients is whether a two-piece prosthesis will provide similar results to its three-piece counterparts. There is limited data in the literature evaluating both the satisfaction rates and surgical outcomes for two-piece IPPs [8,9]. The aim of this study was to evaluate patient and partner satisfaction, device reliability, and complications in patients who underwent two-piece IPP implantation.

## Materials and methods

Study design and patients

This study was approved by the ethics committee of Istanbul Medipol University (approval number: 2021-127) and conducted in accordance with the principles of the Declaration of Helsinki.

The data of patients who underwent penile prosthesis implantation in a tertiary academic center between January 2015 and December 2018 were retrospectively analyzed. Patients who underwent two-piece IPP implantation (AMS Ambicor, American Medical System, Minnetonka, MN, USA) were enrolled in the study. Secondary procedures due to failed primary penile prosthesis surgery were excluded. Patient demographics and clinical data, including age, body mass index (BMI), comorbidities, previous surgery, ED etiology, and duration of ED, were recorded. All patients were evaluated in terms of detailed history, physical examination, laboratory tests (serum glucose, lipid profile, and testosterone values), and the five-item version of the International Index of Erectile Function. All the patients were refractory to conservative treatment (medical, intracavernous self-injection, or vacuum devices), and end-stage ED was confirmed with penile Doppler ultrasonography.

Surgical procedure

All penile prosthesis candidates were informed about the surgical procedure and penile implant types. In the decision-making process prior to surgery, a total of 22 patients agreed to have a two-piece IPP implanted based on their choice, insurance coverage, or socioeconomic and medical status.

After obtaining informed consent, the AMS Ambicor IPP placement was performed. The patient was placed in the supine position. The genital area was shaved before the operation and scrubbed with povidone-iodine for 10 minutes. Antibiotic prophylaxis with vancomycin and ceftriaxone was performed before the procedure. A 16-French urethral catheter was inserted. The corpora cavernosa were reached through a 3- to 5-cm penoscrotal incision. Following bilateral 2-cm corporotomies, corpora cavernosa were dilated up to 13 mm with sharp and blunt dissections. The cavities were irrigated with gentamicin solution. The length of the cavities was measured using a Furlow instrument, and an appropriately sized implant with or without a rear tip extender was placed. The corporotomies were closed with a 2-0 polyglactin (Vicryl) suture. The pump was placed in the scrotal subdartos pouch. Following a hydraulic test, the implant was left semi-inflated. The procedure was completed by suturing the subcutaneous tissue and skin. On postoperative day 1, the urethral catheter was removed, and the patients were discharged with oral ciprofloxacin therapy. The size of the implanted penile prosthesis, complications, duration of operation, and hospital stay were recorded for all patients.

Follow-up

Six weeks after the implantation, the patients were instructed on how to use the prosthesis, and sexual intercourse was allowed. Thereafter, the patients were followed up in the third month, sixth month, and then once a year. Face-to-face and phone interviews were administered to determine the patients’ and their partners’ satisfaction rates with penile prosthesis implantation and surgical outcomes. The modified Erectile Dysfunction Inventory of Treatment Satisfaction (EDITS) questionnaire was used to evaluate the couples’ satisfaction [10]. This questionnaire was developed to evaluate patient and partner satisfaction, the degree to which the prosthesis meets patient expectations, its suitability for continuous use and ease of use, and the impact of treatment on sexual confidence. In addition to the EDITS, the patients were administered a non-validated questionnaire containing four items requiring “yes” or “no” answers (Table 1).

**Table 1:** Domains of the non-validated questionnaire administered to the patients

Questions	Answers
1. Did your prosthesis cause a decrease in penile length?	Yes/no
2. Would you recommend the same procedure to another patient?	Yes/no
3. Do you have satisfying feelings during sexual intercourse?	Yes/no
4. Do you achieve orgasms?	Yes/no

The data on the penile size were only based on the patients’ perceptions. The sexual intercourse frequency of the patients was also recorded.

## Results

A total of 22 patients were included in the study. The mean patient age, mean BMI, and mean partner age were 57.95 ± 6.16 (range: 40-67) years, 27 ± 2.62 kg/m^2^, and 58.12 ± 6.66 years, respectively. The mean ED period was 5.33 ± 2.16 years, and the etiologies of ED were radical pelvic surgery (41%), diabetes mellitus (37%), and vascular disorders (22%). According to the penile Doppler ultrasound findings, 12 (55%) patients had signs of arterial insufficiency, six (27%) had veno-occlusive dysfunction, and four (18%) had combined pathologies.

The mean operative time and mean length of implanted cylinders and rear tip extenders were 102 ± 29 minutes and 18.1 ± 1.68 cm, respectively. The mean postoperative hospital stay and mean postoperative follow-up duration were 1.8 ± 0.66 days and 29.04 ± 14.48 months, respectively. Complications occurred in two patients (9%) due to mechanical device failure in one and prosthesis infection in the other. The patient with a history of retropubic radical prostatectomy due to prostate cancer developed a prosthetic infection three months after implantation. The implant was removed, and antibiotic therapy was applied. The patient with mechanical failure underwent successful revision surgery with the same type of implant. The demographic and clinical characteristics of the patients are presented in Table 2.

**Table 2:** Clinical characteristics of the patients *Continuous variables are presented as mean ± SD.

Variables*	
Patients (number)	22
Patient age (years)	57.95 ± 6.16
Partner age (years)	58.12 ± 6.66
Body mass index (kg/m^2^)	27 ± 2.62
Duration of erectile dysfunction (years)	5.33 ± 2.16
Etiologies of erectile dysfunction (%)	
Radical pelvic surgery	41%
Diabetes mellitus	37%
Vascular disorders	22%
Penile Doppler US findings (%)	
Arterial insufficiency	55%
Veno-occlusive dysfunction	27%
Combined pathologies	18%
Operative time (minutes)	102 ± 29
Length of implanted cylinders and rear tip extenders (cm)	18.1 ± 1.68
Hospital stay (days)	1.8 ± 0.66
Follow-up period (months)	29.04 ± 14.48
Complications (number (%))	2 (9%)
Device infection	1 (4.5%)
Mechanical failure	1 (4.5%)

All 22 patients completed the modified EDITS and non-validated questionnaire. The overall responses to the modified EDITS are given in Table 3. Of the patients, 73% were very satisfied or partially satisfied with the two-piece IPP and stated that it completely or partially met their expectations, and 82% of the patients found the penile prosthesis very or partially easy to use and suitable for continuous use, while 73% reported that they felt fully or partially confident concerning their competence in sexual intercourse. Of the partners, 59% were partially or very satisfied with the implants.

**Table 3:** Results obtained using the modified Erectile Dysfunction Inventory of Treatment Satisfaction (EDITS) for Ambicor penile prostheses

Questions	Answers	Number (%)
1. Overall, how satisfied are you with your penile prosthesis?	Very satisfied	14 (64%)
	Partially satisfied	2 (9%)
	Dissatisfied	6 (27%)
2. To what extent did penile prosthesis meet your expectations?	Completely	13 (59%)
	Partially	3 (14%)
	Not at all	6 (27%)
3. Is the penile prosthesis appropriate for continuous use?	Very appropriate	11 (50%)
	Partially appropriate	7 (32%)
	Inappropriate	4 (18%)
4. Is it easy for you to use the penile prosthesis?	Very easy	11 (50%)
	Partially easy	7 (32%)
	Not easy	4 (18%)
5. How confident do you feel about your competence in sexual intercourse?	Very confident	13 (59%)
	Partially confident	3 (14%)
	Not confident	6 (27%)
6. Are you generally satisfied with your partner’s penile prosthesis?	Very satisfied	9 (41%)
	Partially satisfied	4 (18%)
	Dissatisfied	9 (41%)

The mean frequency of sexual intercourse expressed by the patients was 2.8 per month. Seventeen (77%) patients reported that they would recommend the prosthesis to a friend, and 15 (68%) reported satisfying feelings during sexual intercourse, while 13 (59%) achieved orgasms. Penile shortening was reported in 16 (73%) patients.

## Discussion

The ultimate goal of penile prosthesis implantation is patient satisfaction, which is influenced by several factors, such as unrealistic expectations, the functionality of the device, complications, and partner acceptance and satisfaction [9]. Therefore, it is mandatory to fully inform couples about the surgical procedure, types of prostheses, and their possible outcomes in order to eliminate unrealistic expectations and improve satisfaction. However, to date, only a few articles have reported these outcomes for Ambicor two-piece IPPs.

In a study evaluating couples’ satisfaction with the Ambicor prosthesis, Levine et al. reported a rate of 90% overall patient satisfaction and 82% partner satisfaction in their series of 131 men over a mean follow-up of 43 months [4]. However, a more recent study by Gentile et al. reported the patient and partner satisfaction rates as 75% and 73%, respectively [9]. The patient and partner satisfaction rates reported in different studies in the literature vary between 75%-90% and 73%-91%, respectively [4,5,8,9]. In the present study, the patient and partner satisfaction rates were slightly lower than in the aforementioned studies. The patients that were dissatisfied with the prostheses stated that they did not have satisfactory feeling in the penis, they could not reach an orgasm, or their expectations were not met. In addition, these patients were less likely to have sexual intercourse than the satisfied patients. Although patients are adequately informed about the procedure and implant, unrealistic expectations can persist and lead to dissatisfaction. Therefore, appropriate counseling and support should be continued for couples after surgery.

Partners should also be included in the decision-making process prior to IPP surgery. Gittens et al. investigated whether female sexual dysfunction affected the patients’ satisfaction following penile prosthesis implantation [11]. They evaluated the partners’ sexual function using the Female Sexual Function Index (FSFI) and found a direct correlation between patient satisfaction and their partners’ FSFI scores. In another study by Vakalopoulos et al., a direct correlation was observed between the EDITS scores of the patients and their partners [12]. In our study, the partners’ satisfaction was lower than that of the patients. One possible explanation for this finding may be sexual dysfunction in the partner, but in the current study, we did not evaluate the sexual function of the partners, which can be considered as one of our limitations. In addition, the partner satisfaction rate was found to be lower than reported in the literature. However, in previous studies, no information is given concerning the factors that may affect sexual function in partners, such as their age and comorbidities. Therefore, further studies should take these variables into consideration when evaluating patient and partner satisfaction.

In studies analyzing the complications of the Ambicor penile prosthesis, the overall complication rates were reported between 2.1% and 9.5% in the general population [4,5,8,9]. In special patient populations, such as transgender individuals undergoing neophallus creation (35.6%) and pelvic organ transplant recipients (22%), higher complication rates were reported [13,14]. The most common complications include device infection, mechanical failure, hematoma, and wound infection, similar to the other types of prosthesis. In our study, the overall complication rate was 9%, seen in the form of device infection and mechanical failure. In the literature, unique complications, such as spontaneous deflation and auto-inflation, are also reported to arise from the design of the Ambicor device [4,5]. However, in our cohort, no patient described spontaneous deflation or auto-inflation.

One of the most feared complications in penile prosthesis surgery is device infection. Although Ambicor is not coated with InhibiZone (a combination of rifampin and minocycline) unlike its three-piece counterparts, its infection rates vary between 0.7% and 4.8% at follow-up times ranging from 27 to 60 months [4,5,9]. Infections occur mostly within the first six months of implantation and are managed with the Mulcahy salvage protocol or explantation. In previous studies, all cases of infection were seen in patients with diabetes or who had previously received pelvic irradiation [4,8]. We observed prosthesis infection in only one patient (4.5%) in the third postoperative month, and the implant was removed. In line with these findings, the infection rates of the Ambicor prosthesis can be considered at an acceptable level. In addition to the mechanical properties of the device, it should be taken into account that various other factors, such as patient selection, surgeon experience, and appropriate prophylaxis, can affect the infection rates.

Mechanical failure is one of the major drawbacks of prostheses. We demonstrated that the mechanical failure rate with the Ambicor prosthesis was 4.5% over a mean follow-up of 29 months. The reported mechanical failure rate of the Ambicor two-piece prosthesis ranges from 0.7% to 6.1% for follow-up durations of two to five years [5,8,9]. In light of these findings, Ambicor remains reliable in terms of mechanical durability; however, it should be taken into consideration that the maximum follow-up of these studies is five years, which can result in underestimating the rate of mechanical failure.

Another major concern for patients undergoing penile prosthesis implantation is the loss of penile length. In many studies evaluating penile length changes following penile prosthesis implantation, it has been reported that many patients perceive a change in penile length, mostly shortening of the penis [4,5,15]. In a study by Levine et al., 51% of the patients reported a change in penile length after two-piece prosthesis implantation, and 82.5% of these patients reported a decrease (mean: 1.6 inches [1-3]) in penile length [4]. Similarly, in the study by Lux et al., 70% of the patients reported a mean 1.5-inch decrease in penile length [5]. In both studies, a lower percent of partners than patients reported a change in penile length. Deveci et al. preoperatively and postoperatively measured the stretched penile length of patients who underwent penile prosthesis implantation and reported that although 71% of the patients perceived a decrease in penile length after surgery, there were no statistically significant differences in penile length [15]. They also noted that the treatment satisfaction scores did not depend on subjective evaluation of penile length loss. Comparable to the reported rates, 73% of the patients in our cohort reported a reduction in penile length after implantation. Yet, most of the patients were satisfied with the treatment. According to our results, perceived penis length loss did not seem to affect satisfaction.

This study has its own limitations. First, the study included a relatively small sample size with a retrospective nature. Second, the partners were not evaluated for sexual dysfunction, and the data on penile length was only based on the patients’ perceptions. These limitations should be considered in further studies.

## Conclusions

In conclusion, the Ambicor two-piece IPP is an effective, reliable, and user-friendly implant with acceptable complication and revision rates and provides a high level of patient and partner satisfaction in selected and fully informed patients. Patients’ medical conditions and expectations, as well as their partners’ preferences, should be taken into consideration during the selection of the prosthesis type.
